# Risk factors associated with the severity of overactive bladder among Syrian patients with type 2 diabetes

**DOI:** 10.1038/s41598-024-67326-w

**Published:** 2024-07-17

**Authors:** Fater A. Khadour, Younes A. Khadour, Weaam Alhatem, Deema Al Barroush

**Affiliations:** 1 Department of Rehabilitation, Tongji Hospital, Tongji Medical College, Huazhong University of Science and Technology, 1095#, Jie-Fang Avenue, Qiaokou District, Wuhan, 430030 Hubei China; 2 Department of Rehabilitation, Faculty of Medicine, Al Baath University, Homs, Syria; 3 Department of Physical Therapy, Cairo University, Cairo, 11835 Egypt

**Keywords:** Overactive bladder, Diabetes mellitus, Syria, Diabetic bladder dysfunction, Risk factors, Diabetes, Diabetes complications, Type 2 diabetes

## Abstract

The prevalence of overactive bladder (OAB) is known to be higher in patients with type 2 diabetes (T2DM). However, few studies have examined specific risk factors contributing to its progression among diabetes mellitus (DM) patients, so this study aimed to investigate the risk factors specific to diabetes mellitus that influence overactive bladder in the Syrian population. This cross-sectional study was conducted at four endocrinology centers in four Syrian provinces: Damascus, Aleppo, Homs, Hama, and Latakia. The study was comprised of patients who had been diagnosed with both T2DM and OAB and had visited these centers from February 2020 to January 2023. The Arabic version of the Overactive Bladder Symptom Score (OABSS) scale was used to categorize the participants based on the severity score into two groups: the mild OAB group and the moderate-severe OAB group. A logistic analysis was conducted to assess the risk factors associated with the OAB among patients with diabetes. Among the 153 patients diagnosed with both DM and OAB, significant distinctions were found between the two groups concerning the severity of overactive bladder, age, duration of diabetes, symptomatic diabetic peripheral neuropathy (DPN), and ankle reflex (*P* < 0.05). Furthermore, a multivariate analysis revealed that age (OR 1.48, 95% CI 0.89–2.19), duration of diabetes (OR 1.94, 95% CI 0.53–2.23), and symptomatic DPN (OR 2.74, 95% CI 1.39–4.13) independently acted as risk factors for the advancement of OAB. The severity of OAB in Syrian patients with diabetes is closely associated with the severity of DM. Factors such as age, duration of diabetes, and symptomatic DPN are independent predictors of the severity of OAB. Patients who experience symptomatic DPN are at an increased risk of developing OAB.

## Introduction

Diabetes mellitus (DM) is a disease characterized by a range of serious complications, including retinopathy, nephropathy, and neuropathy. One specific type of neuropathy associated with diabetes is diabetic neuropathy, which can be further classified as diabetic peripheral neuropathy (DPN) or autonomic neuropathy^1,2^. Among the various manifestations of autonomic neuropathy, diabetic bladder dysfunction is notable, presenting as involvement of the lower urinary tract or as overactive bladder (OAB) symptoms.

Following a joint report by the International Urogynecological Association (IUGA) and the International Continence Society (ICS) in 2010, the OAB syndrome is defined as the presence of urinary urgency, often accompanied by frequent nocturia, with or without urgency urinary incontinence (UUI), in the absence of urinary tract infection or other identifiable pathological conditions^3^. The presence of DM and its associated complications significantly impact the quality of life for affected individuals. However, the extent to which different complications of DM affect quality of life can vary. A cross-sectional study conducted in 2017, which involved 1025 individuals with diabetes, revealed that those with DM combined with OAB experienced the most significant impairments in both physical and mental aspects of their quality of life when compared to individuals with other DM complications^4^.

As well as the relationship between metabolic syndrome and the progression of OAB has been explored. A systematic review and meta-analysis that included 13 observational studies with 24,698 participants found that metabolic syndrome was associated with an increased risk of OAB^5^. Another study followed a cohort of older adults for 5 years and found that individuals with metabolic syndrome had a significantly higher incidence of OAB compared to those without metabolic syndrome; the underlying mechanisms suggested for the occurrence of OAB involve insulin resistance, inflammation, and oxidative stress associated with metabolic syndrome^6^. Additionally, a cross-sectional study reported a positive correlation between the number of metabolic syndrome components and the severity of overactive bladder symptoms^7^. This indicates that addressing the individual components of metabolic syndrome, such as obesity, hypertension, and dyslipidaemia, may be important in managing the progression of OAB.

The occurrence of OAB in individuals with diabetes can be attributed to various factors, including systemic inflammation, diabetic angiopathy, and neuropathy. These factors collectively contribute to chronic ischemia of both the bladder^8^ and the central nervous system^9^. Consequently, early identification of the risk factors associated with the severity of OAB in diabetic patients would greatly enhance the clinical management of these two conditions. However, the majority of recent studies have primarily focused on exploring the relationship between conditions such as urinary incontinence and diabetic bladder dysfunction^10–15^. To the best of our knowledge, there is a lack of reports specifically investigating the risk factors associated with the severity of OAB in the diabetic population of Syria. Consequently, we conducted a cross-sectional study to collect comprehensive clinical data and investigate the risk factors associated with the severity of OAB in patients diagnosed with type 2 diabetes mellitus (T2DM). We analyzed the severity of OAB by considering various factors, including age, gender, and diabetic-related variables such as body mass index (BMI), duration of DM, glycosylated hemoglobin A1c (HbA1c) levels, DM treatment, and DM-related complications.

## Methods

### Enrolment of the patients

This cross-sectional study was conducted at four endocrinology centers in four Syrian provinces: Damascus, Aleppo, Homs, Hama, and Latakia. One center was selected from each province. The study included patients who had both T2DM and OAB and had visited these centers between February 2020 and January 2023. The diagnosis of T2DM was based on the guidelines provided by the World Health Organization (WHO) and similar previous articles. The diagnosis was confirmed by a physician specializing in endocrinology at our center. Patients with fasting plasma glucose levels ≥ 7 mmol/L or 2-h postprandial glucose levels ≥ 11.1 mmol/L were diagnosed as having diabetes^16–19^. The diagnosis of OAB was confirmed using the Overactive Bladder Symptom Score (OABSS) scoring system with the following diagnostic criteria: patients with urgency scores of OABSS ≥ 2 and an overall score ≥ 3^20^.

Patients with certain diseases were excluded from this study, including those with pelvic organ prolapse, spinal cord injury (e.g., spinal cord injury, lumbar rigidity, spinal bifida, etc.), neuropathy (e.g., Parkinson’s disease, multiple sclerosis, etc.), previous bladder and urethral lesions, history of urinary tract tuberculosis, history of major pelvic or bladder surgery, urinary tract infections, and gestational diabetes. The study received approval from the Ethical Committee of the Al Baath University Institutional Review Board. A consent letter, indicated by the Consent Letter–IRB 2023168-S, was obtained from the participants.

### Demographic and medical information

The medical information used in this study was taken from the patients' electronic medical files as follows: (1) demographic and clinical data including BMI, duration of DM, gender, height, weight, HbA1c level, DM treatment, ankle reflexes, and age; (2) comorbidities including, heart disease, diabetic nephropathy, cerebrovascular disease, hypertension, hyperlipidaemia, and DPN.

### Examination symptoms of DPN

All patients in the study received a thorough clinical examination encompassing pinprick, temperature, vibration perception (using a 128-Hz tuning fork), 10-g monofilament pressure sensation at the distal halluces, and assessment of ankle reflexes. If the examination results yielded positive findings, it strongly indicated the presence of DPN. Patients who exhibited positive results in multiple clinical tests and demonstrated typical symptomatology, such as numbness, paresthesia, burning pain, dysesthesia, and muscle cramps, were diagnosed with symptomatic DPN ^21^.

### Examination of the severity of OAB

The Arabic version of the OABSS questionnaire was used to assess the severity of OAB accurately. It consists of four items that assess symptoms related to overactive bladder: daytime frequency, nighttime frequency, urgency, and urgency incontinence. The total scores on the OABSS range from 0 to 15, with higher scores indicating more severe symptoms. In this study, patients were categorized based on the severity of their symptoms as follows: mild (OABSS 3–5), moderate (OABSS 6–11), and severe (OABSS 12–15)^20,22^. The patients in the study were classified into two groups: the mild group (OABSS 3–5) and the moderate-severe group (OABSS 6–15).

### Statistical analysis

Statistical analysis was conducted using various data, including general factors (age, gender, height, weight, BMI), factors related to diabetes (HbA1c, DM treatment, and DM duration), factors of DPN (ankle reflex and vibratory sensibility), and items in overactive bladder (OABSS). Continuous variables are presented as mean ± standard error (SE), while categorical variables are presented as numbers and percentages. The independent sample t-test was performed to identify differences between continuous variables, and Pearson's chi-square analysis was used for categorical variables. Multiple logistic regression analysis was applied to assess the contribution of risk factors to the mild OAB and moderate-severe OAB groups. Statistical analysis was conducted using SPSS version 16 (SPSS Inc, Chicago). A p-value less than 0.05 was considered statistically significant.

### Ethics approval and consent to participate

The Ethical Committee approved this study in the Al Baath University Institutional Review Board Consent Letter—IRB 2023168-S; all procedures were conducted under the ethical principles outlined in the 1964 Declaration of Helsinki and subsequent revisions. Patients were informed of the study's purpose and procedures. In addition, the participants provided written informed consent to participate in this study.

## Results

This study initially included 208 individuals with T2DM occurring between 2020 and 2024. However, during the screening process, 55 cases were excluded for various reasons. These reasons encompassed 8 cases with spinal injury,13 cases with Parkinson’s, 5 cases with stroke, 5 cases with persistent urinary infection, 14 cases with prostate problems, and 10 cases with pelvic organ complications. Finally, 153 individuals with T2DM were included in the statistical analysis. The age range of the participants was 41 to 52 years, with a median age of 59.3 years. Their durations of DM varied from 7 to 31 years, with an average duration of 13.5 years. The patients' BMI ranged from 18.2 to 34.4 kg/m2, with a mean BMI of 22.9 kg/${\text{m}}^{2}$. The biochemical tests conducted for HbA1c yielded results ranging from 5 to 13%, with an average value of 6.2%. Among the subjects, 44 patients received oral hypoglycemia drugs, 83 were administered insulin treatment, and 26 patients were prescribed a combination of both treatments. Furthermore, 21.6 of patients used Beta-3 adrenergic agonists to manage their OAB, while 13.1 of the patients used antimuscarinic (anticholinergic) medications; in terms of disease history and complications, 33 patients presented with heart disease. Additionally, 74 patients had hypertension, and 88 patients were diagnosed with hyperlipidaemia. Importantly, 67.79% of the patients exhibited DPN, with 50.98% reporting obvious nervous symptoms and 16.99% being asymptomatic Table 1.

**Table 1:** Demographic and biochemical data of diabetic patients with OAB (n = 153).

Parameter	Value
Age (y)	59.3 ± 13.2
Sex	
Male	59 (38.56%)
Female	94 (61.43%)
Body mass index (kg/${\text{m}}^{2}$ )	22.9 ± 4.1
Diabetes duration (y)	13.5 ± 7.2
HbA1c	6.2 ± 1.5
HbA1c (%)	7.86 ± 1.68
Total cholesterol (mg/dl)	162.35 ± 40.6
HDL-C (mg/dl)	45.32 ± 13.16
LDL-C (mg/dl)	102.26 ± 33.15
Triglyceride (mg/dl)	142.61 ± 73.53
FPG (mg/dl)	144.37 ± 52.19
Medication for controlling the diabetes	
Oral drug	44 (28.75%)
Insulin	83 (54.24%)
Oral drug + insulin	26 (16.99%)
History of using medication of diabetes	
Antimuscarinic (anticholinergic) medications	20 (13.07)
Beta-3 adrenergic agonists	33 (21.56)
Antimuscarinic (anticholinergic) + Beta-3 adrenergic agonists	14 (9.15)
DPN	71 (50.32%)
With symptoms	26 (16.99%)
Without symptoms	78 (50.98%)
History of hypertension	74 (48.36%)
History of hyperlipidaemia	88 (57.51%)
History of heart disease	33 (21.56%)
OABSS	
Mild OAB	89 (58.16%)
Moderate OAB	53 (34.64%)
Severe OAB	11 (7.18%)

Value represents mean ± standard error or numbers (%). Abbreviations: DPN, diabetic peripheral neuropathy; HbA1c, glycosylated haemoglobin A1c; LDL-C, Low-density lipoprotein cholesterol; HDL-C, High-density lipoprotein cholesterol; FPG, Fasting plasma glucose.OAB, overactive bladder; OABSS, overactive bladder symptom score.

Based on the OABSS, it was found that 58.16% (89 out of 153) of patients exhibited mild symptoms of OAB. Those with moderate OAB accounted for 34.64% (53 out of 153), while the remaining 7.18% (11 out of 153) constituted the severe OAB group. Notably, significant differences were observed among the groups in terms of age (< 60/ ≥ 60 years), duration of diabetes (< 10/ ≥ 10 years), bilateral ankle reflex (presence or absence), and symptomatic DPN (presence or absence) Table 2.

**Table 2:** Univariate analysis of variance of mild OAB and moderate‐severe OAB in diabetic patients.

Variable	Mild OAB	Moderate‐Severe OAB	OR (95% CI)	*P* value
Sex				
Male	39 (66.11%)	20 (33.89%)		
Female	68 (72.34%)	26 (27.66%)	2.62 (1.42‐2.94)	0.341
Age (y)				
< 60	36 (66.64%)	18 (33.36%)		
≥ 60	62 (62.61%)	37 (37.39%)	3.41 (1.73‐3.12)	0.031*
BMI (kg/${\text{m}}^{2}$)				
< 25	82 (67.75%)	39 (32.25%)		
25	21 (65.62%)	11 (34.37%)	1.31 (0.82‐1.26)	0.082
Diabetic duration (y)				
< 10	42 (68.86%)	19 (31.14%)		
≥ 10	61 (66.31%)	31 (33.69%)	2.03 (1.45‐2.47)	0.041*
HbA1c (%)				
< 7	81 (66.39%)	41 (33.61%)		
≥ 7	22 (70.96%)	9 (29.03%)	1.45 (0.92‐1.32)	0.241
Total cholesterol (mg/dl)				
< 170	66 (61.68%)	37 (34.32%)		
≥ 170	21 (42.02%)	29 (57.98%)	2.69 (1.79‐2.59)	0.094
HDL-C (mg/dl)				
< 70	51 (54.83%)	42 (42.16%)		
≥ 70	18 (32.14%)	38 (67.86%)	3.66 (1.93‐4.83)	0.371
LDL-C (mg/dl)				
< 100	43 (47.76%)	47 (52.24%)		
≥ 100	29 (46.03%)	34 (53.97%)	1.63 (0.59‐1.83)	0.175
Triglyceride (mg/dl)				
< 150	63 (62.37%)	38 (37.63%)		
≥ 150	22 (42.30%)	30 (57.70%)	2.06 (0.95‐2.69)	0.084
FPG (mg/dl)				
< 130	71 (67.61%)	34 (32.39%)		
≥ 130	27 (56.24%)	21 43.76%)	3.69 (1.85‐4.79)	0.152
Treatment				
Oral drug	28 (70.02%)	12 (29.98%)	0.58 (0.31‐1.14)	
Insulin	62 (76.54%)	19 (23.46%)	1.24 (0.74‐1.35)	0.251
Oral drug + insulin	22 (68.75%)	10 (31.25%)	2.15 (0.93‐2.29)	0.358
Ankle reflexes				
Present	71 (77.17%)	21 (22.83%)		
Absent	45 (73.77%)	16 (26.23%)	2.13 (1.36‐2.32)	0.0327*
Vibration perception				
Present	41 (66.13%)	21 (33.87%)		
Absent	33 (67.34%)	16 (32.66%)	1.24 (0.83‐1.53)	0.471
Symptomatic DPN				
Present	53 (60.92%)	34 (39.08%)		
Absent	49 (74.24%)	17 (25.76%)	2.57 (1.14‐3.67)	0.005**
Hypertension history				
With	68 (70.14%)	31 (29.86%)		
Without	39 (72.24%)	15 (27.76%)	0.89 (0.53‐1.53)	0.452
Hyperlipidaemia history				
With	69 (70.41%)	29 (29.59%)		
Without	36 (65.62%)	19 (34.38%)	1.02 (0.58‐1.24)	0.357
Heart disease history				
With	41 (80.39%)	10 (19.61%)		
Without	69 (67.62%)	33 (32.38%)	1.31 (0.94‐1.72)	0.428

*P < .05,

**P < .01 represent statistical difference.

(%). Abbreviations: DPN, diabetic peripheral neuropathy; HbA1c, glycosylated haemoglobin A1c; LDL-C, Low-density lipoprotein cholesterol; HDL-C, High-density lipoprotein cholesterol; FPG, Fasting plasma glucose.OAB, overactive bladder;

The multivariate analysis revealed that the duration of diabetes, the presence or absence of symptomatic DPN, and age were identified as independent risk factors associated with the progression of OAB. Among these factors, the presence or absence of symptomatic DPN was found to be the most significant, with an odds ratio of 2.74 (95% CI 1.39–4.13) (Table 3).

**Table 3:** Multivariate analysis of variance of mild OAB and moderate‐ severe OAB in diabetic patients.

Variable	Mild OAB	Moderate‐Severe OAB	OR (95% CI)	*P* value
Age (y)				
< 60	36 (66.64%)	18 (33.36%)		
≥ 60	62 (62.61%)	37 (37.39%)	1.48 (0.89‐2.19)	0.041*
Diabetic duration (y)				
< 10	42 (68.86%)	19 (31.14%)		
≥ 10	61 (66.31%)	31 (33.69%)	1.94 (1.53‐3.23)	0.032*
Ankle reflexes				
Present	71 (77.17%)	21 (22.83%)		
Absent	45 (73.77%)	16 (26.23%)	1.12 (0.75‐1.79)	0.097
Symptomatic DPN				
Present	53 (60.92%)	34 (39.08%)		
Absent	49 (74.24%)	17 (25.76%)	2.74 (1.39‐4.13)	0.029*

*P < .05,

**P < .01 represent statistical difference.

## Discussion

According to estimates from 2022, the overall prevalence of diabetes mellitus among the adult population in Syria has reached 21.4%^23^. Diabetic bladder dysfunction is the most common and bothersome chronic complication affecting the lower urinary tract in individuals with diabetes, with a prevalence of 43.0% to 87.0% among diabetic patients^24^. However, there has been a lack of sufficient research conducted on diabetic bladder dysfunction.

This study found significant differences in age, duration of DM, symptomatic DPN, and ankle reflexes between the mild OAB group and the moderate-severe OAB group. The multivariate analysis identified age, duration of diabetes, and symptomatic DPN as independent risk factors for the severity of OAB. A previous study also demonstrated a close association between the development and severity of DPN with the duration of DM and age^25^. Therefore, it can be inferred that as the duration of DM and age increase, the likelihood of developing DPN also increases, leading to worsening OAB symptoms. Moreover, experiments have shown that the pathogenesis of DPN is linked to factors such as hyperglycemia, glucotoxicity, impaired insulin signaling, and other risk factors that contribute to structural changes in the nervous system^26^. DPN is primarily characterized by demyelination, axonal degeneration, and fiber loss^27^. The primary causes of DPN are peripheral and autonomic neuropathy, which affect sensory afferent pathways. This leads to a gradual onset of impaired bladder sensation and ultimately results in decreased detrusor contractility and bladder dysfunction. This may explain the association between DPN and OAB. However, further investigations are necessary to explore other potential causes and factors involved in this relationship.

The ankle reflex, despite showing a positive association with the severity of OAB, was not identified as an independent risk factor. However, it has previously been considered a reliable indicator of diabetic neuropathy in asymptomatic individuals^28^. Although it exhibits a high sensitivity (91.5%) and specificity (67.4%)^29^, it is not suitable for assessing the severity of neuropathy. Nonetheless, for patients who present with symptomatic DPN, the ankle reflex may serve as a threshold point for recognizing the severity of OAB. When diagnosing symptomatic DPN, it is important to consider the signs and symptoms along with conducting quantitative peripheral sensory tests. The presence of typical symptoms such as numbness, paresthesia, burning pain, dysesthesia, and muscle cramps should be carefully observed during the diagnostic process. It is conceivable that patients whose neuropathic condition has progressed beyond the threshold of symptom development are more likely to experience severe neuropathy compared to those who are still in the pre-symptomatic stage ^21^. The presence of neuropathic symptoms is indicative of severe diabetes mellitus, and therefore, the ankle reflex can be utilized as a parameter to reflect the progression of OAB.

The evaluation of bladder function typically relies on urodynamic assessment, which involves using a double-lumen cystometry catheter and a balloon rectal catheter. This procedure is not convenient, especially for diabetic patients with mild symptoms of OAB. It is typically reserved for diabetic patients experiencing difficulties in urination or those requiring surgery due to urinary retention. Since this examination is invasive, it is not suitable for OAB patients as it may worsen the symptoms of OAB. Additionally, it has the potential to cause irreversible changes to bladder function, and if the diagnosis is delayed, it can hinder effective OAB treatment. Therefore, exploring the risk factors associated with OAB is crucial to facilitate early detection and enhance the management of this condition. This approach can help mitigate the need for invasive procedures and improve the overall treatment outcomes for individuals with OAB.

Furthermore, previous studies have shown that diabetic patients with OAB demonstrate increased bladder wall thickness on ultrasonographic assessment compared to diabetic patients without OAB symptoms. This increased bladder wall thickness is thought to result from chronic inflammatory changes and detrusor muscle hypertrophy associated with diabetic bladder dysfunction. The urodynamic findings in these patients often reveal detrusor overactivity, reduced bladder compliance, and impaired bladder sensation—all of which can contribute to the development of OAB symptoms. These urodynamic and ultrasonographic findings support the close relationship between diabetes and the development of overactive bladder in this patient population^26,27,30^.

This study did not specifically stratify the results by gender, as we focused on investigating the risk factors for OAB severity in the overall T2DM population. These are attributed to the fact that there are some male patients with OAB after benign prostatic hyperplasia (BPH) or after transurethral resection of the prostate (TURP). The troublesome symptoms after transurethral resection/incision of the prostate (TUR-P/TUI-P) are similar to those of OAB, although their underlying pathophysiology may be completely different^31^. The evidence from research studies suggests that obstruction at the bladder outlet can lead to morphological changes in epithelial and detrusor muscle cells^32,33^. It not only alters its ultrastructure but also causes interference within neuronal networking. Such a transformation, resulting in decreased capacity, low contractility, poor compliance, and hyperactive bladder, may persist even after surgical relief of BPH.

Our results demonstrated that 44 patients received oral hypoglycemia drugs, 83 were administered insulin treatment, and 26 were prescribed a combination of both treatments. These results are in line with previous studies^2,13^. Furthermore, 33 of the patients used Beta-3 adrenergic agonists to manage their OAB, while 20 of them used antimuscarinic (anticholinergic) medications. The management of OAB in patients with T2DM has evolved alongside the advancements in the treatment of diabetes itself^34,35^. In the past, the primary focus for patients with T2DM was on glycemic control through lifestyle modifications and traditional antidiabetic medications, such as metformin, sulfonylureas, and insulin^36,37^. However, the potential impact of these therapies on lower urinary tract function and OAB symptoms was not well understood.

As the pathophysiology of OAB in the context of T2DM has improved, the healthcare community has recognized the importance of a more holistic approach to managing these co-occurring conditions^38,39^. This has led to the introduction of newer antidiabetic agents, such as GLP-1 agonists and SGLT-2 inhibitors, which have shown promising effects in improving OAB symptoms in patients with T2DM^40,41^. Additionally, the use of antimuscarinic medications and β3-adrenoceptor agonists, specifically developed for the treatment of OAB, has become more prevalent in this patient population^42,43^. These targeted OAB therapies have demonstrated the ability to alleviate OABSS in individuals with T2DM. Therefore, it is crucial to consider the evolving landscape of medical treatments for T2DM and OAB when investigating the relationship between these conditions and their impact on OABSS. Accounting for the specific pharmacotherapies used by the study population can provide valuable insights into the complex interplay between diabetes management and lower urinary tract function.

This study has several limitations that should be considered. Firstly, it was conducted at only four centers, which may limit the generalizability of the findings to a broader population of diabetic patients. Therefore, caution should be exercised when extrapolating the results to other settings or populations. Secondly, the study focused on elderly patients who may have had neurogenic bladder due to various underlying factors such as benign prostatic hyperplasia, urethral stricture, urinary tract infections, and other conditions. However, the study did not investigate or evaluate these potential contributing factors. Although transrectal ultrasounds and urine flow rate examinations were performed to minimize research errors, the presence of these confounding factors could still influence the results and should be taken into account.

In conclusion, this study suggests that in individuals with type 2 diabetes and OAB, the severity of OAB is associated with symptomatic DPN, duration of diabetes, and age. Notably, symptomatic NPD was found to exacerbate OAB symptoms. However, due to the limitations mentioned, it is important to interpret these results with caution. Further research, including larger and more diverse studies, is needed to validate these findings and explore additional factors that may contribute to developing OAB in diabetic patients. When evaluating diabetic patients with OAB, it is recommended to monitor the severity of OAB, symptomatic diabetic peripheral neuropathy, duration of diabetes, and age as important parameters in clinical practice.

## Data Availability

The datasets generated and/or analyzed during the current study are not publicly available due [to maintain the privacy of the patients participating in the study] but are available from the corresponding author on reasonable request.
